# Roles of the AMPA receptor subunit GluA1 but not GluA2 in synaptic potentiation and activation of ERK in the anterior cingulate cortex

**DOI:** 10.1186/1744-8069-5-46

**Published:** 2009-08-10

**Authors:** Hiroki Toyoda, Ming-Gao Zhao, Bettina Ulzhöfer, Long-Jun Wu, Hui Xu, Peter H Seeburg, Rolf Sprengel, Rohini Kuner, Min Zhuo

**Affiliations:** 1 Department of Physiology, Faculty of Medicine, University of Toronto, University of Toronto Centre for the Study of Pain, 1 King's College Circle, Toronto, Ontario M5S 1A8, Canada; 2 Institute for Pharmacology, University of Heidelberg, Im Neuenheimer Feld 366, 69120 Heidelberg, Germany; 3 Department of Molecular Neurobiology, Max-Planck Institute for Medical Research, Jahnstrasse 29, 69120 Heidelberg, Germany; 4 Department of Brain and Cognitive Sciences, Seoul National University, Seoul 151-746, Korea

## Abstract

Cortical areas including the anterior cingulate cortex (ACC) are important for pain and pleasure. Recent studies using genetic and physiological approaches have demonstrated that the investigation of basic mechanism for long-term potentiation (LTP) in the ACC may reveal key cellular and molecular mechanisms for chronic pain in the cortex. Glutamate N-methyl D-aspartate (NMDA) receptors in the ACC are critical for the induction of LTP, including both NR2A and NR2B subunits. However, cellular and molecular mechanisms for the expression of ACC LTP have been less investigated. Here, we report that the α-amino-3-hydroxy-5-methyl-4-isoxazolepropionic acid (AMPA) receptor subunit, GluA1 but not GluA2 contributes to LTP in the ACC using genetic manipulated mice lacking GluA1 or GluA2 gene. Furthermore, GluA1 knockout mice showed decreased extracellular signal-regulated kinase (ERK) phosphorylation in the ACC in inflammatory pain models *in vivo*. Our results demonstrate that AMPA receptor subunit GluA1 is a key mechanism for the expression of ACC LTP and inflammation-induced long-term plastic changes in the ACC.

## Introduction

Activity-dependent synaptic plasticity in the central nervous system (CNS) has been proposed to contribute to major brain functions, including memory, chronic pain and drug addiction [1-4]. Long-term potentiation (LTP) is a major form of synaptic plasticity, and the enhancement of synaptic transmission in central regions related to sensory transmission and perception is believed to be a key cellular mechanism for chronic pain [2,5]. The anterior cingulate cortex (ACC) is a major cortical area that is believed to contribute to injury-related unpleasantness and memory in animal models of pain and memory [6-9]. Activation of postsynaptic glutamate NMDA receptor by different stimulation protocols triggers LTP in pyramidal neurons of the ACC [10-13]. Calcium-dependent intracellular signaling proteins, including AC1 (adenylyl cyclase subtype 1), ERK (extracellular signal-related kinase) and CaMKIV (calmodulin-dependent protein kinase IV) are found to contribute to ACC LTP [11,14-16].

Glutamatergic AMPA (α amino-3-hydroxy-5-methylisoxazole-4-propionic acid) receptors mediate the majority of fast excitatory synaptic transmission in the brain, including the ACC region [17-19]. In the forebrain areas, AMPA receptors are heteromeric complexes assembled from mainly GluA1 and GluA2 [20]. The other two subunits of AMPA receptor, GluA3 and GluA4 express at relative lower levels [21,22]. According to the new subunit nomenclature recommended by the International Union of Basic and Clinical Pharmacology (IUPHAR), these AMPA subunits are renamed as GluA1, GluA2, GluA3 and GluA4 [23]. The requirement of different AMPA subtype receptors for LTP is likely to be regional-, development-dependent [24-27]. For example, in the hippocampal CA1 region, GluA1 is required for LTP in adult but not juvenile animals [25,27]. Furthermore, LTP in the cerebellum require GluA2 subunit [28]. It is also important to note that not all cortical LTP share the similar mechanisms. In the somatosensory cortex, Frey et al reported that GluA1 is not required for the LTP in the layer II/III barrel cortex [24]. However, in the ACC, using postsynaptic injection of different peptide inhibitors Toyoda et al found that GluA1 contribute to LTP in the layer II/III pyramidal neurons [29]. One possible difference between these experiments is the methods of pharmacological and genetic approaches, in addition to the different cortical region investigated.

In the present study, we performed whole-cell patch-clamp recordings from ACC and somatosensory cortex (SSC) neurons to test the role of AMPA receptor subunits for long-term synaptic plasticity by using mice lacking the genes for GluA1 or GluA2. Furthermore, we analyzed ERK1/2 phosphorylation in these cortical regions by using mouse models of inflammation. We observed that the AMPA receptor subunits, GluA1 and GluA2 differentially contribute to LTP in the ACC and SSC. Moreover, GluA1 knockout mice showed the decreased cortical activation of ERK1/2 *in vivo*. Our results provide strong evidence that the induction of cortical plasticity and persist pain could be triggered by GluA1-mediated, ERK-dependent signaling pathway.

## Results

### GluA1 subunits are involved in synaptic potentiation in the ACC

**Figure 1:**
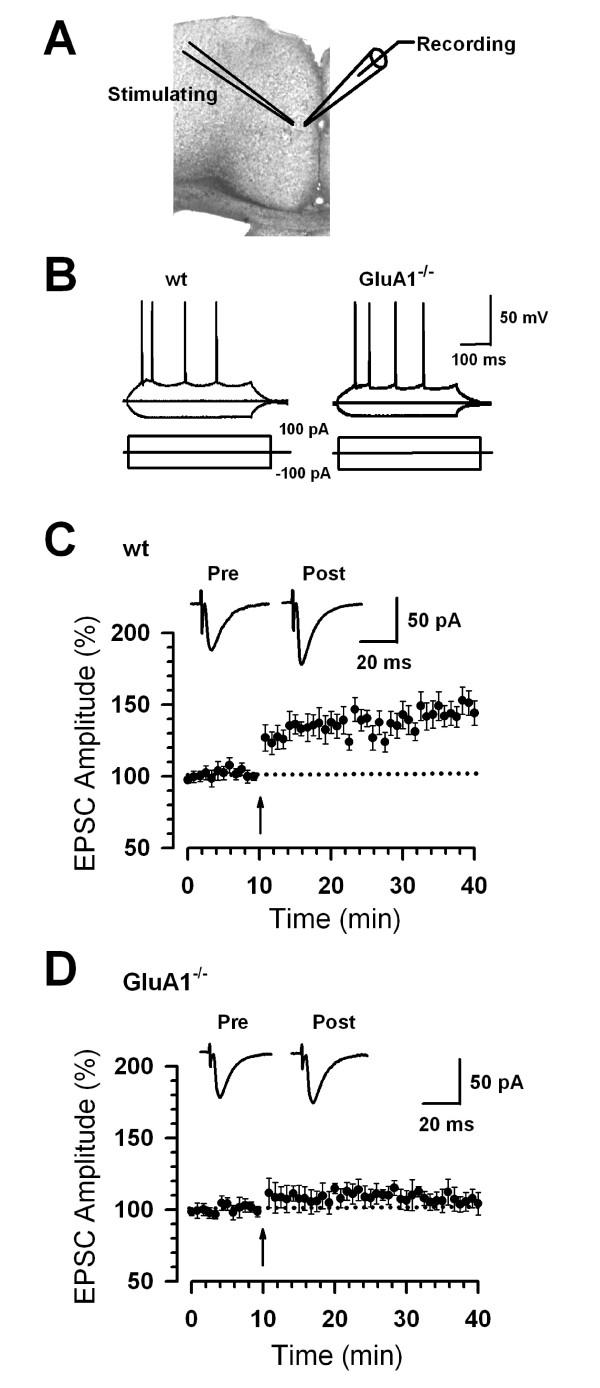
**Abolishment of cingulate potentiation in *GluA1*^-/- ^mice**. (A) Diagram of a slice showing the placement of a whole-cell patch recording and a stimulation electrode in the ACC. (B) These traces showing typical voltage responses to current injections of -100, 0, and 100 pA in ACC neurons from WT and *GluA1*^-/- ^mice. (C) LTP was induced in ACC pyramidal neurons in WT mice (n = 13 slices/6 mice). (D) LTP was lost in ACC pyramidal neurons in *GluA1*^-/- ^mice (n = 8 slices/6 mice). (C-D) The insets show averages of five EPSCs at baseline responses and 30 min after the pairing procedure (arrow). The dashed line indicates the mean basal synaptic responses.

It is evident that injuries trigger a series of plastic changes in pain-related cortical regions including the ACC [2,30-32]. Thus, the investigation of the molecular and cellular mechanisms regarding ACC plasticity provides insights into how the ACC processes and modulates sensory information. To reveal the roles of GluA1 and GluA2 subunits for synaptic potentiation in the ACC, we took genetic approach by using GluA1 and GluA2 knockout mice (*GluA1*^-/- ^and *GluA2*^-/-^, respectively) in the present study. We performed whole-cell patch-clamp recordings from visually identified pyramidal neurons in layer II/III of the ACC slices from *GluA1*^-/- ^mice and their wild-type (WT) mice. Fast excitatory postsynaptic currents (EPSCs) were obtained by delivering focal electrical stimulation to layer V (see Fig. 1A). In addition to visual identification, we confirmed that the recordings were performed from cortical pyramidal cells by injecting depolarizing currents into the neuron (Fig. 1B). Intrinsic membrane properties and action potential firing were compared between WT and *GluA1*^-/- ^mice. No significant differences in passive or active intrinsic properties between neurons from WT (n = 11) and *GluA1*^-/- ^mice (n = 10) were detected (*t*-test, *P *> 0.05). Table 1 summarizes the measurement of resting membrane potential, input resistance and action potential characteristics in WT and *GluA1*^-/- ^mice.

**Table 1:** Summary of basic electrophysiological properties for ACC neurons in wild-type and GluA1 and GluA2 knockout mice

	***WT***	***GluA1***^-/-^	***WTCD1***	***GluA2***^-/-^
**Resting membrane Potential (mV)**	68.3 ± 0.8	68.0 ± 1.0	67.8 ± 1.0	68.5 ± 0.8
**Input resistance (MΩ)**	113.5 ± 5.4	112.4 ± 5.0	130.3 ± 6.9	154.8 ± 14.0
**Frequency (Hz)**	4.6 ± 0.5	4.0 ± 0.4	4.6 ± 0.3	4.4 ± 0.5
**AP amplitude (mV)**	93.8 ± 5.5	94.8 ± 5.0	102.3 ± 1.5	103.5 ± 1.9
**AP Half-width (ms)**	2.3 ± 0.2	2.3 ± 0.3	1.9 ± 0.1	1.8 ± 0.1
**AHP amplitude (mV)**	10.0 ± 2.0	8.8 ± 1.6	7.7 ± 0.5	7.0 ± 0.7

Next, we studied the synaptic potentiation in WT and *GluA1*^-/- ^mice. We used the typical LTP induction paradigm to trigger LTP in ACC slices, which contained presynaptic 80 pulses at 2 Hz with postsynaptic depolarization at +30 mV (referred to as the pairing training) [13]. We induced LTP within 12 minutes after establishing the whole-cell configuration to avoid washout of intracellular contents that are critical for the establishment of synaptic plasticity [13]. LTP was induced by pairing training which produced a significant, long-lasting potentiation of synaptic responses in slices of WT mice (35 min to 40 min after the conditioning, mean 146.0 ± 8.3% of baseline, n = 13 slices/6 mice, *t*-test; *P *< 0.001 compared with baseline responses before the pairing training, Fig. 1C). By contrast, synaptic potentiation was absent in slices from *GluA1*^-/- ^mice (106.8 ± 7.2%, n = 8 slices/6 mice, *t*-test; *P *> 0.05 compared with baseline responses, Fig. 1D). These results provide the first genetic evidence that GluA1 is critical for LTP in the ACC of adult mice.

### AMPA receptor-mediated EPSCs are reduced in *GluA1*^-/- ^mice

**Figure 2:**
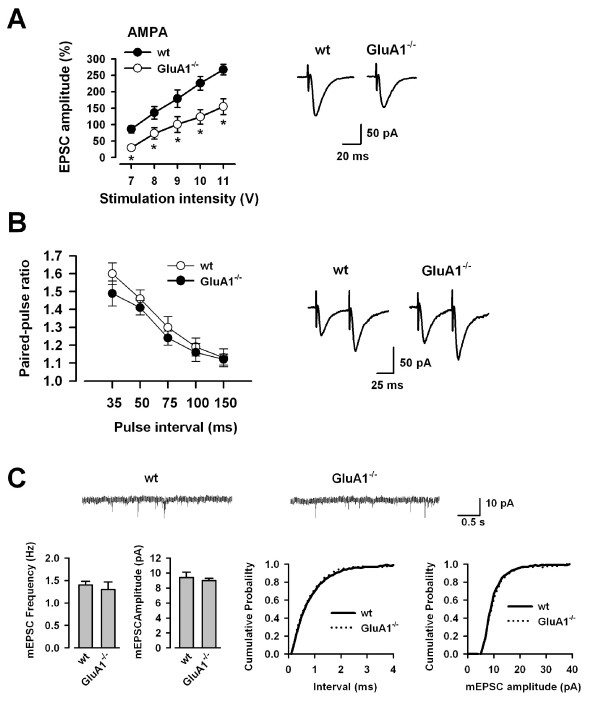
**Reduced AMPA receptor-mediated EPSCs in *GluA1*^-/- ^mice**. (A) Input-output relationships for AMPA receptor-mediated EPSCs in WT (n = 7) and *GluA1*^-/- ^(n = 6) mice (left). * *P *< 0.05 compared with WT mice. Traces showing averages of five AMPA receptor-mediated EPSCs with input stimulation at 9 V (right). (B) Paired-pulse facilitaion (PPF) did not differ in WT (n = 13) and *GluA1*^-/- ^(n = 9) mice (left). Sample traces of PPF recorded from WT and *GluA1*^-/- ^mice at the 50 ms interval (right). (C) Traces of mEPSCs recorded from WT and *GluA1*^-/- ^mice (Top). Summary results showing the frequency and the amplitude of mEPSCs in ACC neurons from WT (n = 13) and *GluA1*^-/- ^(n = 9) mice (Bottom, left). Cumulative probability plot showing the distribution of the inter-event interval and the frequency in WT (n = 13) and *GluA1*^-/- ^(n = 9) mice (Bottom, right).

Considering the abolishment of synaptic potentiation in the ACC of *GluA1*^-/- ^mice, we decided to examine if basal synaptic transmission may be altered in *GluA1*^-/- ^mice. First, we analyzed AMPA receptor-mediated EPSCs evoked by various stimulus intensities in the presence of the NMDA receptor blocker AP-5 (50 μM). The input-output relationship of AMPA receptor-mediated EPSCs in *GluA1*^-/- ^mice (n = 6) was significantly reduced as compared with WT mice (n = 7; Fig. 2A, left). The rise time and the decay time in AMPA receptor-mediated EPSCs with input stimulation at 9 V showed no significant difference in *GluA1*^-/- ^(rise time, 3.3 ± 0.2 ms; decay time, 17.9 ± 1.0 ms, n = 6) mice in comparison with WT mice (rise time, 3.2 ± 0.2 ms; decay time, 17.6 ± 1.6 ms, n = 7) (Fig. 2A, right). These findings indicate that GluA1 contributes to basal synaptic transmission in the ACC.

We next examined paired-pulse facilitation (PPF) to test whether presynaptic function was altered in *GluA1*^-/- ^mice. There was no difference in PPF ratio in *GluA1*^-/- ^mice (n = 12) compared with WT mice (n = 13) (Fig. 2B); indicating that basic presynaptic release properties is likely intact in *GluA1*^-/- ^mice. We further examined mEPSCs from WT and *GluA1*^-/- ^mice and found no significant differences in either the frequency (1.4 ± 0.1 vs 1.3 ± 0.2 Hz, *t*-test; *P *> 0.05) or the amplitude (9.4 ± 0.7 vs 9.0 ± 0.3 pA, *t*-test; *P *> 0.05) in ACC neurons of WT (n = 13) vs *GluA1*^-/- ^mice (n = 9) (Fig. 2C). The rise time and the decay time in mEPSCs showed no significant difference in *GluA1*^-/- ^(rise time, 1.9 ± 0.2 ms; decay time, 11.4 ± 0.4 ms, n = 9) mice in comparison with WT mice (rise time, 2.2 ± 0.1 ms; decay time, 12.0 ± 0.4 ms, n = 13) (Fig. 2C). These results suggest that the reduction of AMPA receptor-mediated EPSCs in *GluA1*^-/- ^mice is unlikely to result from presynaptic changes.

### NMDA receptor-mediated EPSCs are intact in *GluA1*^-/- ^mice

**Figure 3:**
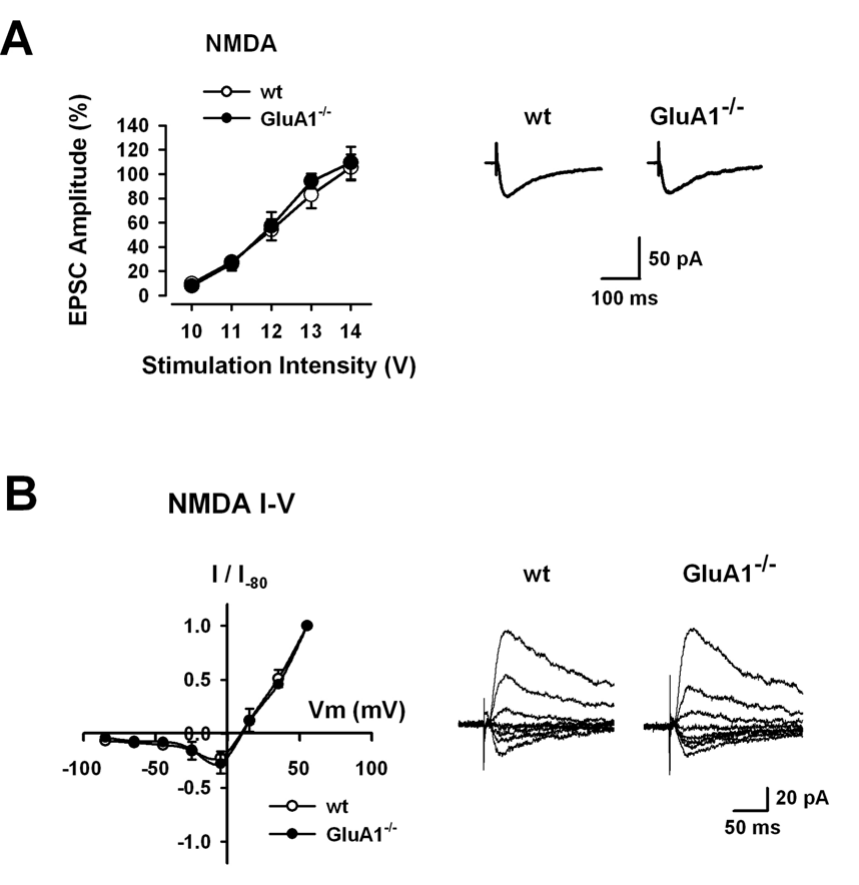
**Intact NMDA receptor-mediated EPSCs in *GluA1*^-/- ^mice**. (A) Input-output relationships for NMDA receptor-mediated EPSCs in WT (n = 6) and *GluA1*^-/- ^(n = 8) mice (left). Traces showing averages of five NMDA receptor-mediated EPSCs with input stimulation at 12 V (right). (B) I-V relationships of NMDA receptor-mediated EPSCs in WT (n = 8) and *GluA1*^-/- ^(n = 8) mice (left). NMDA receptor-mediated EPSCs recorded at holding potentials from -85 mV to +55 mV in WT and *GluA1*^-/- ^mice (right).

NMDA receptors are critical for the induction of LTP in the ACC [13]. To test the possibility that the deletion of GluA1 subunit affect the induction of LTP by inhibiting NMDA receptor-mediated currents, we first examined the NMDA receptor-mediated EPSCs evoked by various stimulus intensities. To record NMDA receptor-mediated EPSCs, we added CNQX (20 μM) and glycine (1 μM) in the recording solution. NMDA receptor-mediated EPSCs in the ACC pyramidal neurons remained unchanged in *GluA1*^-/- ^(n = 8) mice in comparison with WT mice (n = 6, Fig. 3A, left). The rise time and decay time in NMDA receptor-mediated EPSCs with input stimulation at 12 V showed no significant difference in *GluA1*^-/- ^(rise time, 19.8 ± 1.2 ms; decay time, 146.9 ± 9.3 ms, n = 8) mice in comparison with WT mice (rise time, 20.7 ± 1.2 ms; decay time, 143.2 ± 7.0 ms, n = 6) (Fig. 3A, right).

We also examined the voltage dependence of NMDA receptor-mediated EPSCs. We recorded the NMDA receptor-mediated EPSCs over a range of membrane potentials from -85 mV to +55 mV. NMDA receptor-mediated EPSCs showed typical rectified I-V relationship with the reversal potential around +5 mV. No difference was found for the I-V relationship of NMDA receptor-mediated EPSCs in WT (n = 8) and *GluA1*^-/- ^(n = 8) mice (Fig. 3B).

### Synaptic potentiation is enhanced in *GluA2*^-/- ^mice

**Figure 4:**
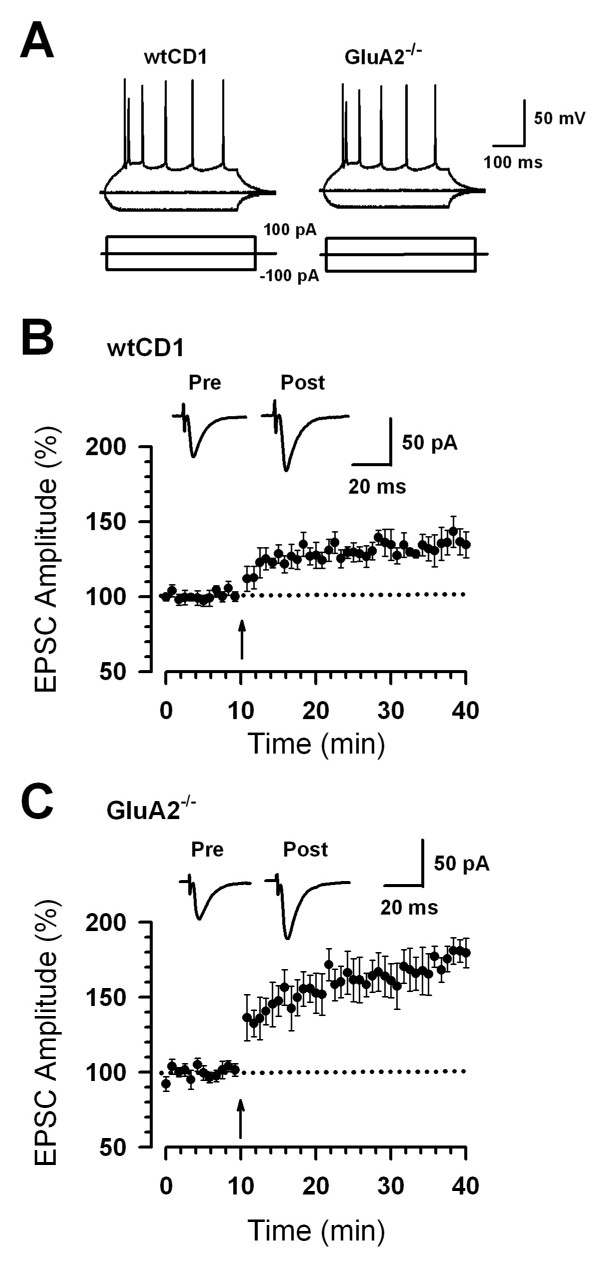
**Enhancement of synaptic potentiation in *GluA2*^-/- ^mice**. (A) These traces show typical voltage responses to current injections of -100, 0, and 100 pA in ACC neurons from WTCD1 and *GluA2*^-/- ^mice. Injection of depolarizing currents into neurons induced repetitive action potentials with frequency adaptation that is typical of the firing pattern of pyramidal neurons. (B) LTP was induced in ACC pyramidal neurons in WTCD1 mice (n = 9 slices/5 mice). (C) LTP was enhanced in ACC pyramidal neurons in *GluA2*^-/- ^mice (n = 8 slices/5 mice) compared with WTCD1 mice. (B-C) The insets show averages of five EPSCs at baseline responses and 30 min after the pairing procedure (arrow). The dashed line indicates the mean basal synaptic responses.

To determine if GluA2 may be also involved in ACC LTP, we performed whole-cell patch-clamp recordings from pyramidal neurons in layer II/III of ACC slices from *GluA2*^-/- ^and their littermate wild-type (WTCD1) mice. There was no significant difference in passive or active intrinsic properties between neurons from WTCD1 (n = 8) and *GluA2*^-/- ^mice (n = 7) (Fig. 4A, see Table 1). We then examined the synaptic potentiation in WTCD1 and *GluA2*^-/- ^mice. Unlike the case observed in *GluA1*^-/- ^mice, the pairing training produced robust LTP in *GluA2*^-/- ^mice (last 5 min mean 177.8 ± 9.8%, n = 8 slices/5 mice; *P *< 0.05 compared with baseline responses, Fig. 4C). The magnitude of synaptic potentiation in *GluA2*^-/- ^mice was significantly greater than that of WTCD1 mice (136.2 ± 10.1% of baseline, n = 9 slices/5 mice, *t*-test; *P *< 0.05 compared with baseline responses, Fig. 4B). These results suggest that GluA1 and GluA2 subunits differentially modulate synaptic potentiation in the ACC of adult mice.

### AMPA receptor-mediated EPSCs are reduced in *GluA2*^-/- ^mice

**Figure 5:**
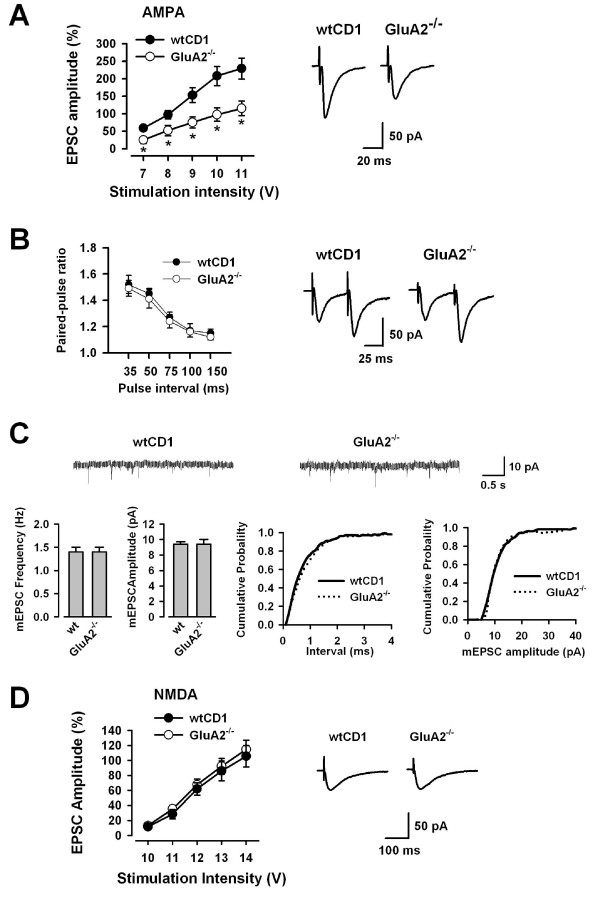
**Reduced AMPA receptor-mediated EPSCs in *GluA2*^-/- ^mice**. (A) Input-output relationships for AMPA receptor-mediated EPSCs in WTCD1 (n = 6) and *GluA2*^-/- ^(n = 6) mice (left). * *P *< 0.05 compared with WT mice. Traces showing averages of five AMPA receptor-mediated EPSCs with input stimulation at 9 V (right). (B) Paired-pulse facilitaion (PPF) did not differ in WTCD1 (n = 7) and *GluA2*^-/- ^(n = 13) mice (left). Sample traces of PPF recorded from WTCD1 and *GluA2*^-/- ^mice at the 50 ms interval (right). (C) Traces of mEPSCs recorded from WTCD1 and *GluA2*^-/- ^mice (Top). Summary results showing the frequency and the amplitude of mEPSCs in ACC neurons from WT (n = 8) and *GluA2*^-/- ^(n = 9) mice (Bottom, left). Cumulative probability plot showing the distribution of the inter-event interval and the frequency in WTCD1 (n = 8) and *GluA1*^-/- ^mice (n = 9) (Bottom, right). (D) Input-output relationships for NMDA receptor-mediated EPSCs in WTCD1 (n = 6) and *GluA1*^-/- ^mice (n = 6) (left). Traces showing averages of five NMDA receptor-mediated EPSCs with input stimulation at 12 V (right).

We also examined AMPA receptor-mediated EPSCs in *GluA2*^-/- ^mice in the presence of 50 μM AP-5. As with *GluA1*^-/- ^mice, *GluA2*^-/- ^mice (n = 6) also showed reduced AMPA receptor-mediated EPSCs at all stimulus intensities compared with WTCD1 mice (n = 6) (Fig. 5A, left). The rise time and decay time in AMPA receptor-mediated EPSCs with input stimulation at 9 V showed no significant difference in *GluA2*^-/- ^(rise time, 3.1 ± 0.1 ms; decay time, 17.6 ± 1.3 ms, n = 6) mice in comparison with WTCD1 mice (rise time, 3.1 ± 0.1 ms; decay time, 17.6 ± 1.3 ms, n = 6) (Fig. 5A, right).

We then examined PPF and found that there was no difference in the level of facilitation in *GluA2*^-/- ^(n = 13) compared with WTCD1 mice (n = 7) (Fig. 5B). We also recorded mEPSCs from WTCD1 and *GluA2*^-/- ^mice. There was no significant difference in either the frequency (1.4 ± 0.1 vs 1.4 ± 0.1 Hz, *P *> 0.05) or the amplitude (9.4 ± 0.3 vs 9.4 ± 0.6 pA, *P *> 0.05) in ACC neurons of WTCD1 (n = 8) vs *GluA2*^-/- ^mice (n = 9) (Fig. 5C). The rise time and the decay time in mEPSCs showed no significant difference in *GluA2*^-/- ^(rise time, 2.2 ± 0.1 ms; decay time, 9.8 ± 0.4 ms, n = 9) mice in comparison with WTCD1 mice (rise time, 2.2 ± 0.1 ms; decay time, 11.0 ± 0.2 ms, n = 8) (Fig. 5C). These results suggest that the reduction of AMPA receptor-mediated EPSCs in *GluA2*^-/- ^mice is unlikely to result from presynaptic changes, similar to the result from *GluA1*^-/- ^mice.

NMDA receptor-mediated EPSCs were examined in the presence of 20 μM CNQX and glycine (1 μM). The NMDA receptor-mediated EPSCs in ACC pyramidal neurons remained unchanged in *GluA2*^-/- ^mice (n = 6) in comparison with WTCD1 mice (n = 6). The rise time and the decay time in NMDA receptor-mediated EPSCs with input stimulation at 12 V showed no significant difference in *GluA2*^-/- ^(rise time, 21.6 ± 1.9 ms, n = 6; decay time, 153.4 ± 8.9 ms, n = 6) mice in comparison with WTCD1 mice (rise time, 19.6 ± 2.0 ms, n = 6; decay time, 149.4 ± 10.9 ms, n = 6) (Fig. 5D). Taken together, these results suggest that AMPA but not NMDA receptor-mediated transmission in *GluA2*^-/- ^mice was also reduced, similar to *GluA1*^-/- ^mice.

### GluA1 and GluA2 subunits differentially modulate synaptic potentiation in somatosensory cortex (SSC)

**Figure 6:**
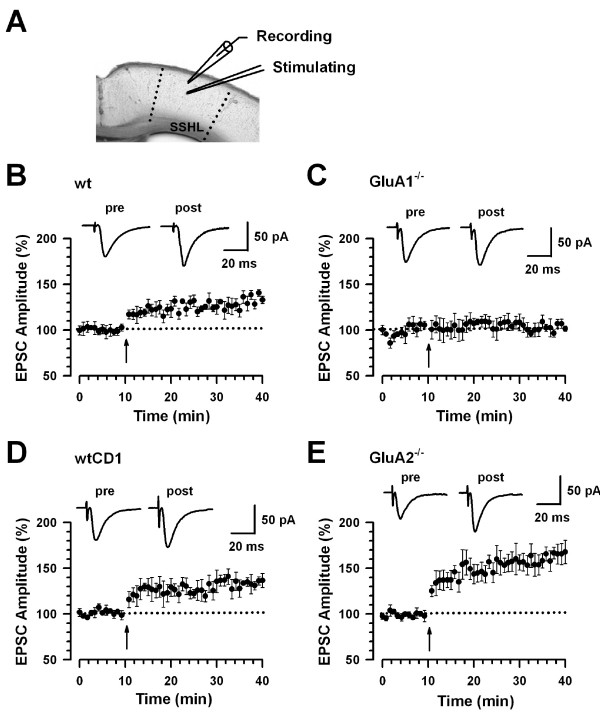
**Synaptic potentiation in the SSHL in GluA1^-/- ^mice**. (A) Diagram of a slice showing the placement of a whole-cell patch recording and a stimulation electrode in the SSHL. (B) LTP was induced in SSHL neurons in WT mice (n = 7 slices/6 mice). (C) LTP was lost in ACC pyramidal neurons from *GluA1*^-/- ^mice (n = 7 slices/5 mice). (D) LTP was induced in SSHL neurons from WTCD1 mice (n = 6 slices/5 mice). (E) LTP was enhanced in SSHL neurons from *GluA2*^-/- ^mice (n = 6 slices/5 mice). (B-E) The insets show averaged of five EPSCs at baseline responses and 30 min after the pairing procedure (arrow). The dashed line indicates the mean basal synaptic responses.

The SSC plays a central role in the processing of sensory inputs, and developmental- or pathology-associated activity-dependent changes in the SSC have been hypothesized to underlie plastic changes in sensory discrimination *in vivo *[33-35]. We therefore addressed the role of GluA1 and GluA2 subunits in sensory activity-related LTP in the SSC. Recordings were performed from pyramidal cells in layer II/III in somatosensory hindlimb cortex (SSHL). We tested synaptic potentiation in SSHL neurons by delivering focal electrical stimulation to layer V (Fig. 6A). In WT mice, the pairing training produced significant synaptic potentiation (134.4 ± 5.4%, n = 7 slices/6 mice, *t*-test; *P *< 0.01 compared to baseline, Fig. 6B). In contrast, synaptic potentiation was lost in slices from *GluA1*^-/- ^mice (102.9 ± 5.1%, n = 7 slices/5 mice, *t*-test; *P *> 0.05 compared with baseline responses, Fig. 6C). We then studied synaptic potentiation in SSHL neurons in *GluA2*^-/- ^mice. As with the ACC, the pairing training produced significant synaptic potentiation in *GluA2*^-/- ^mice (160.1 ± 10.1%, n = 6 slices/5 mice; *P *< 0.05 compared with baseline responses, Fig. 6E) as well as in WTCD1 mice (134.2 ± 5.6% of baseline, n = 6 slices/5 mice, *t*-test; *P *< 0.05 compared with baseline responses, Fig. 6D). The magnitude of synaptic potentiation was significantly enhanced in *GluA2*^-/- ^mice (134.2 ± 5.6% for WTCD1 versus 160.1 ± 10.1% for *GluA2*^-/-^, *t*-test; *P *< 0.05). These results suggest that the GluA1 and GluA2 subunits differently modulate synaptic plasticity in the SSC, consistent with the ACC.

### Inflammatory pain is associated with activation of ERK1/2 in cortical neurons

**Figure 7:**
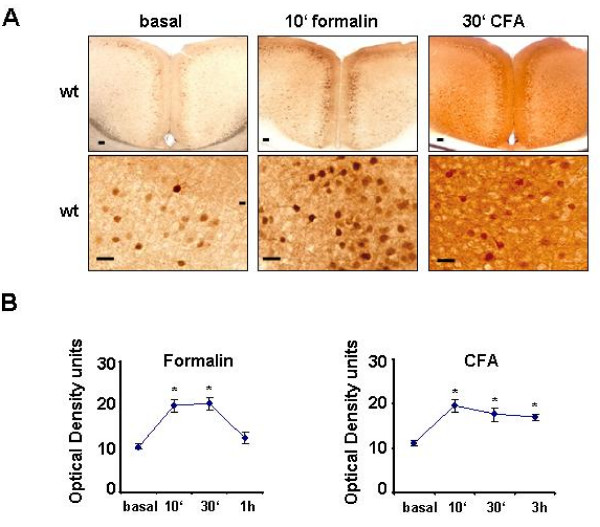
**ERK phosphorylation as an indicator of synaptic plasticity in the ACC in mouse models of inflammatory pain**. (A) Typical examples of increase in immunoreactivity for phospho-ERK1/2 in ACC of WT mice at 10 minutes (min) following intraplantar (ipl.) injection of formalin or 30 min following intraplantar injection of complete Freund's adjuvant (CFA). (B) Analysis of the time-course and quantification of nociceptive activity-induced ERK phosphorylation in ACC via densitometric analysis of immunostained sections.

What do these ex-vivo slice findings mean in the context of plasticity in the cortex *in vivo*? LTP in the ACC is proposed to be a key cellular model [30,36-38] and ACC LTP is likely contributing to both the early cortical changes in the ACC as well as plastic changes in the ACC after the injury [2]. We therefore chose mouse models of persistent nociceptive activity to address mechanisms of synaptic plasticity in the ACC *in vivo*. Recent work from our lab as well as others showed that ACC ERK is activated after peripheral inflammation [39,40]. Considering the fact that ERK activity is required for ACC LTP [14], it is conceivable that activity-dependent LTP may contribute to activation of ERK1/2 in the ACC in animal models of persistent pain. To address the cortical levels of activated (phosphorylated) ERK1/2 in inflammatory pain states, we utilized mouse models based upon intraplantar injection of 1% formalin or complete Freund's adjuvant (CFA) in the mouse hindpaw. The plantar formalin test is a measure of rapid sensitization of nociceptive pathways and involves formalin-evoked nocifensive responses in two phases: an acute phase I (0–10 minutes following injection), which is caused by direct activation of nociceptors by formalin and a subacute phase II (15–50 minutes), which is believed to involve spinal, cortical as well as peripheral sensitization mechanisms. In the basal (naïve) state, only a few neurons in the ACC showed immunoreactivity for phosphorylated ERK1/2 (pERK1/2) (Fig. 7A). At 10 minutes after formalin injection, a significant increase was observed in the immunoreactivity for pERK over neurons of the ACC, as judged by densitometry (*P *< 0.05 as compared with basal levels; see Fig. 7A for typical examples and Fig. 7B for quantitative summary). Whereas only a few neurons demonstrated strong immunoreactivity over the neuronal somata in the basal state, following formalin injection, prominent immunoreactivity was observed in the dendrites as well as the somata of a large number of neurons (Fig. 7A, higher magnification). Formalin-induced increase in pERK immunoreactivity was sustained at 30 minutes after injection, and returned to basal levels at 60 min after injection, after the phase II response had subsided (Fig. 7B).

To address whether long-lasting hypersensitivity evoked by peripheral inflammation is associated with cortical activation of ERK1/2, we analyzed pERK1/2 immunoreactivity at 10 min, 30 min and 3 hours after hindpaw injection of CFA. Injection of CFA in the hindpaw triggered inflammation within minutes and led to a rapid and long-lasting hyperalgesia to thermal and mechanical stimuli (Fig. 7B). Concurrent to the course of hyperalgesia, CFA evoked a rapid and long-lasting increase in pERK immunoreactivity over neurons of the ACC (see Fig. 7A and Fig. 7B for summary; *P *< 0.05 as compared with basal levels). In particular, intense immunoreactivity was observed in the dendrites and neuropil after CFA administration (Fig. 7A, higher magnification).

### Nociceptive-activity induced cortical ERK1/2 activation in AMPA receptor subunit knockout mice

**Figure 8:**
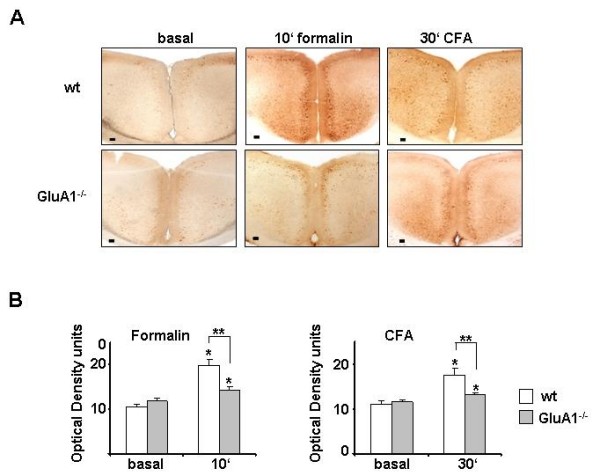
**ERK phosphorylation in the ACC with inflammatory-relayed pain in WT and GluA1^-/- ^mice**. (A) ERK immunoreactivity of control, and 10 min after formalin injection or 30 min after CFA injection in control (WT, top panel) and *GluA1*^-/- ^mice (lower panel). (B) Quantitative summary of ERK immunoreactivity in control and *GluA1*^-/- ^mice.

Given the importance of both ERK and GluA1-containing AMPA receptors in plasticity phenomena in the ACC, we asked whether AMPA receptors could act upstream of nociceptive activity-evoked activation of ERK1/2 in the cortex. Phosphorylation of ERK1/2 in neurons of the ACC induced by intraplantar injection of either formalin or CFA was significantly decreased in *GluA1*^-/- ^mice in comparison with their WT mice (see Fig. 8A for typical example and Fig. 8B for summary of densitometric analysis). In particular, dendrites of cortical neurons were rarely immunoreactive for pERK1/2 in formalin- or CFA-injected *GluA1*^-/- ^mice. In contrast, nociceptive activity-evoked ERK1/2 phosphorylation of ERK1/2 remained intact in the ACC of *GluA2*^-/- ^mice, as compared to WTCD1 mice (not shown).

## Discussion

In the present study, we have demonstrated that AMPA receptor GluA1 subunit contributes to the expression of LTP in pain-related ACC region. This finding is consistent with our previous report using postsynaptic injection of AMPA receptor GluA1 interfering peptide inhibitor [29]. Furthermore, *GluA1*^-/- ^mice showed the significant decrease in cortical ERK activation in two *in vivo *animal models of inflammatory pain. Thus, AMPA GluA1-ERK pathway is likely to play an important role in cortical synaptic plasticity, which would be essential for higher brain functions such as persistent pain and related memory and emotional responses. Future experiments are clearly needed to explore the roles of GluA1-ERK in different forms of chronic pain.

### ACC and chronic pain

Cumulative evidence from both human and animal studies demonstrates that the ACC is important for pain-related perception and chronic pain. It has been demonstrated that local lesions of the medial frontal cortex, including the ACC, reduced acute nociceptive responses, injury-related aversive behaviors, and chronic pain in rodents [32,41,42]. Electrophysiological recordings showed that ACC neurons responded to peripheral noxious stimuli, and neuroimaging studies in humans have further confirmed these observations and showed that the ACC, together with other cortical structures, were activated by acute noxious stimuli, psychological pain, and social pain (see [2]). Cellular and molecular mechanisms for long-term plastic changes in ACC neurons have been investigated using genetic and pharmacological approaches, and several key signaling proteins or molecules have been identified including calcium-stimulated adenylyl cyclase (AC) 1, AC8, NMDA receptor NR2B subunit [7-9,43,44]. After persistent inflammation, the expression of NMDA NR2B receptors in the ACC was up-regulated with the enhanced behavioral responses [44], consistent with the increased inflammation-related persistent pain in NR2B forebrain overexpression mice [7]. We also found the attenuated behavioral sensitization in various chronic pain models in mice lacking AC1 and AC8 [8,43]. Moreover, enhancements of not only presynaptic enhancements of glutamate release but also postsynaptic glutamate receptor-mediated responses in the ACC were mediated by cAMP signaling pathway [8,9,45]. Recent studies using animal models of inflammatory and neuropathic pain reported that the ERK signaling pathway in the ACC contributes to both induction and expression of chronic pain [39,40]. In the current study, we further extended the molecular and cellular mechanisms relating the long-term plastic changes in ACC neurons by demonstrating that GluA1-ERK pathway may play an important role in early changes within the ACC. This provides the first evidence that GluA1-ERK pathway plays vital roles in activity-dependent synaptic plasticity in the ACC.

### Molecular mechanisms of LTP induction in the ACC

The molecular and cellular mechanisms of synaptic potentiation in the ACC are beginning to be elucidated by pharmacological and genetic studies. The neuronal activity triggered by LTP-inducing stimuli increases the release of glutamate in the cingulate synapses. The activation of NMDA receptors including NR2A and NR2B subunits and L-type voltage-gated calcium channels (L-VDCCs) causes an increase in postsynaptic calcium in dendritic spines [11,13]. Calcium influx via NMDA receptors and L-VDCCs plays a key role for triggering biological processes that lead to LTP in the ACC. Postsynaptic calcium then binds to calmodulin and triggers various intracellular protein kinases and phosphatases [46]. Calmodulin target proteins, such as Ca^2+^/calmodulin-dependent protein kinases (PKC, CaMKII and CaMKIV), calmodulin-activated ACs (AC1 and 8), and the calmodulin-activated phosphatase calcineurin, are known to be important for synaptic plasticity in the hippocampus [1,47]. Among them, we found that activation of AC1 and CaMKIV is essential for the induction of LTP in the ACC [11,15]. As the downstream target of AC1, cAMP-dependent protein kinase (PKA) may activate MEK and ERK/MAPK. The role of MAPK cascade in the induction of cingulate LTP has been documented in a previous study [14], which showed that activation of MAPK including ERK, JNK and p38 is critical for the induction of cingulate LTP. In addition, activated ERK/MAPK likely has multiple targets including cAMP response element binding protein (CREB) that is required for long-term synaptic changes in neurons [15].

### GluA1 and GluA2 subunits in cortical LTP

Several studies suggest that these receptor subunits may play distinct roles in the regulation of AMPA receptor trafficking and synaptic plasticity. The GluA1 subunit is required for NMDA receptor-dependent synaptic delivery of AMPA receptors, a process thought to be responsible for the activity-dependent delivery of AMPA receptors during LTP [48-53]. We have recently examined the role of GluA1 subunit using pharmacological approaches and found that the GluA1 subunit C-terminal peptide analog Pep1-TGL blocked the induction of cingulate LTP [29]. Thus, in the ACC of adult mice, the interaction between the C terminus of GluA1 and PDZ domain proteins is required for the induction of LTP. Our results in this paper show that the ACC and SSHL slices prepared from adult *GluA1*^-/- ^mice failed to elicit LTP. This result is consistent with the previous reports that LTP was impaired in *GluA1*^-/- ^mice in the hippocampus [27,54]. The postsynaptic Ca^2+ ^influx via NMDA receptors activates the CaMKII and this also activates Ras and ERK [53,55]. This signaling cascade is suggested to be involved in GluA1-dependent LTP [55]. In contrast, GluA2/GluA3 receptors may continually replace preexisting synaptic AMPA receptors in an activity-independent manner [56-59]. The GluA2/GluA3 receptors may play a complementary role in the constitutive delivery pathway via GluA2-mediated interaction with N-ethylmaleimide-sensitive fusion protein (NSF) and class II PDZ domain proteins [50]. The functional significance of GluA2 and GluA3 in synaptic plasticity has been extensively studied in CA1 hippocampus neurons [60,61]. We here show that synaptic potentiation is enhanced in ACC and SSHL in *GluA2*^-/- ^mice. Thus, our experiments using GluA1/2 KO mice suggest that the AMPA receptor subunits, GluA1 and GluA2, act differentially in ACC LTP.

### Activity-dependent ERK activation *in vivo*

An interesting finding of this paper is that both, ERK1/2 and the GluA1 subunit are important in activity-dependent changes in the ACC *in vivo*. Peripheral injuries are known to lead to a sustained phosphorylation and activation of ERK1/2 in sensory neurons of the dorsal root ganglia as well as in spinal dorsal neurons [62-64]. Here we report a rapid and sustained phosphorylation of ERK1/2 in neurons of the ACC induced by persistent activation of nociceptors following CFA injection. These observations, coupled to our previous finding that ERK activation is necessary for LTP in the ACC [14] strongly suggests that ERK activation is an important step in triggering long-lasting potentiation of cortical neurons, which is critically linked with induction and maintenance of chronic pain. Interestingly, *GluA1*^-/- ^mice demonstrated a diminished activation of cortical ERK in responses to persistent nociception *in vivo *and a loss of cortical potentiation ex-vivo. This is consistent with our previous findings that *GluA1*^-/- ^mice demonstrate diminished behavioral hyperalgesia in models of inflammatory pain [62]. Thus, the composition of cortical as well as spinal AMPA receptors may be a key determinant for pathological pain states which are triggered by persistent activation of nociceptors in inflamed or injured tissue.

In summary, we demonstrate the strong ex-vivo as well as in-vivo evidence that the ERK-GluA1 pathway is essential for synaptic plasticity in pain-related cortical regions. This study might further improve our understanding of cellular and molecular mechanisms of cortical plasticity and help to identify new targets for the treatment of patients with chronic pain.

## Materials and methods

### Genetically-modified mice

Null mutant mice for genes encoding GluA1 (*gria1*) and GluA2 (*gria2*) have been described previously [27,65]. GluA1^-/- ^mice were crossed back into the C57BL/6 strain, and the GluA2^-/- ^mice were crossed back into the CD1 strain, each for more than eight generations. GluA gene knockout mice and control littermates were obtained by interbreeding heterozygous mice.

### Slice preparation

The Animal Care and Use Committee of University of Toronto approved the mouse protocols. Coronal brain slices (300 μM) containing the anterior cingulate cortex (ACC) and somatosensory hindlimb cortex (SSHL) from six- to eight-week-old GluA gene knockout mice and their control littermates were prepared using standard methods [13]. Slices were transferred to a submerged recovery chamber with oxygenated (95% O_2 _and 5% CO_2_) artificial cerebrospinal fluid (ACSF) containing (in mM: 124 NaCl, 2.5 KCl, 2 CaCl_2_, 1 MgSO_4_, 25 NaHCO_3_, 1 NaH_2_PO_4_, 10 glucose) at room temperature for at least 1 h.

### Whole-cell recordings

Experiments were performed in a recording chamber on the stage of an Axioskop 2FS microscope with infrared DIC optics for visualization of whole-cell patch clamp recording. Excitatory postsynaptic currents (EPSCs) were recorded from layer II/III neurons with an Axon 200B amplifier (Molecular Devices, CA) and the stimulations were delivered by a bipolar tungsten stimulating electrode placed in layer V of the ACC and SSHL. EPSCs were induced by repetitive stimulations at 0.02 Hz and neurons were voltage clamped at -70 mV. The recording pipettes (3–5 MΩ) were filled with solution containing (mM). 145 K-gluconate, 5 NaCl, 1 MgCl_2_, 0.2 EGTA, 10 HEPES, 2 Mg-ATP, and 0.1 Na_3_-GTP (adjusted to pH 7.2 with KOH). After obtaining stable EPSCs for 10 min, the LTP induction paradigm was used within 12 min after establishing the whole-cell configuration to prevent wash out effect on LTP induction [66]. The LTP-inducing protocol involved paired presynaptic 80 pulses at 2 Hz with postsynaptic depolarization at +30 mV (referred to as pairing training). The NMDA receptor-mediated component of EPSCs was pharmacologically isolated in ACSF containing: CNQX (20 μM), glycine (1 μM) and picrotoxin (100 μM). The patch electrodes contained (in mM) 102 cesium gluconate, 5 TEA chloride, 3.7 NaCl, 11 BAPTA, 0.2 EGTA, 20 HEPES, 2 MgATP, 0.3 NaGTP, and 5 QX-314 chloride (adjusted to pH 7.2 with CsOH). Neurons were voltage clamped at -30 mV and NMDA receptor-mediated EPSCs were evoked at 0.05 Hz. Picrotoxin (100 μM) was always present to block GABA_A _receptor-mediated inhibitory currents. Access resistance was 15–30 MΩ and monitored throughout the experiment. Data were discarded if access resistance changed more than 15% during an experiment. Rise times were determined between 10 and 90% of the peak amplitude of the evoked and miniature EPSC. Decay times were measured between 90 and 10% of peak amplitude.

### Pharmacological inhibitors

All chemicals and drugs were obtained from Sigma (St. Louis, MO), except for QX-314, which was from Tocris Cookson (Ellisville, MO).

### Immunohistochemistry

Mice were perfused with 0.1 M phosphate buffer saline and 4% paraformaldehyde (PFA) and brains were isolated and post-fixed for up to 16 h in 4% PFA. Free-floating sections (100 μm, vibratome), were processed for immunohistochemistry anti-phospho-ERK1/2 antibody (Cell Signaling Inc., 1: 1000 dilution) as described in details previously [62]. Densitometric analysis of pERK immunoreactivity was performed over ACC and SSHL using the Cell Explorer Software (Serva, Heidelbeg, Germany) in at least 3–4 sections per mouse from at least 3 mice per treatment group as described in details previously [62].

Furthermore, the following antibodies were used: rabbit polyclonal anti-GluA2/3 and anti-GluA1 antisera (Chemicon International, Hofheim, Germany). Mice were perfused transcardially with 4% paraformaldehyde (PFA) and spinal cords, brains or dorsal root ganglia were extracted and postfixed overnight in 4% PFA. Immunohistochemistry was performed on vibratome sections (50 μm) or cryosections (20 μm) using standard reagents and protocols (Vector Laboratories, Burlingame, USA). Sections from treatment groups to be compared were stained and photographed together and care was taken to ensure that the staining reaction was within the linear range. Brightfield images were taken under similar illumination conditions.

### Data analyses

Results were analyzed by t-test, paired t-test, or two-way ANOVA followed by post-hoc student-Newman-Keuls test to identify significant differences. Data are expressed as mean ± S.E.M. In all cases, *P *< 0.05 was considered statistically significant.

## Abbreviations

AC: adenylyl cyclase; ACC: anterior cingulate cortex; ACSF: artificial cerebrospinal fluid; AMPA: α-amino-3-hydroxy-5-methyl-4-isoxazolepropionic acid; CaMK: calmodulin-dependent protein kinase; CFA: complete Freund's adjuvant; CREB: cAMP response element binding protein; EPSC: excitatory postsynaptic current; ERK: extracellular signal-related kinase; LTD: long-term depression; LTP: long-term potentiation; NSF: N-ethylmaleimide-sensitive fusion protein; NMDA: N-methyl D-aspartate receptor; pERK: phosphorylated ERK; PKA: cAMP-dependent protein kinase; PPF: paired-pulse facilitation; SSC: somatosensory cortex; SSHL: somatosensory hindlimb cortex.

## Competing interests

The authors declare that they have no competing interests.

## Authors' contributions

HT, MGZ and UH are responsible for performance of experiments and writing the manuscript. WLJ and XH are responsible for performance of experiments. SPH and SR are responsible for experimental design. RK and MZ are responsible for experimental design and writing the manuscript. All authors read and approved the final manuscript.
